# A Feasible Method Applied to One-Bath Process of Wool/Acrylic Blended Fabrics with Novel Heterocyclic Reactive Dyes and Application Properties of Dyed Textiles

**DOI:** 10.3390/polym12020285

**Published:** 2020-02-01

**Authors:** Meihui Wang, Xianfeng Wang, Chong Guo, Tao Zhao, Wenyao Li

**Affiliations:** 1 College of Chemistry, Chemical Engineering and Biotechnology, Donghua University, Shanghai 201620, China; 2150561@mail.dhu.edu.cn (M.W.); Wxfrain1102@outlook.com (X.W.); 15071473653@163.com (C.G.); 2 Key Laboratory of Science and Technology of Eco-Textile, Ministry of Education, Donghua University, Shanghai 201620, China; 3 College of Material Engineering, Shanghai University of Engineering Science, Shanghai 201620, China

**Keywords:** heterocyclic reactive dyes, wool/acrylic blended fabrics, cationic group, one-bath process, levelling properties, anti-ultraviolet, antibacterial activities

## Abstract

Reactive dyes containing cationic groups have great potentiality as novel dyes, which can be applicable to one-bath dyeing of wool/acrylic blended fabrics. In this work, four novel heterocyclic reactive dyes containing cationic groups were designed by using m-aminophenyltrimethylammonium salt or *N*-(2-aminoethyl) pyridinium chloride salt as cationic groups, *N, N*-diethyl-1,3-benzenediamine as a coupling component, 2-amino-6-methoxybenzothiazole, 2-aminobenzothiazole or 3-amino-5-nitrobenzoisothiazole as diazo components. These dyes based on benzothiazole derivative chromophores not only showed beautiful color, including blue-green and fuchsia, but also had larger tinctorial strength with a high molar extinction coefficient, further reducing the dosage of dyes to achieve same color depth. Factors affecting the dyeability on fabrics, such as pH value, dyeing temperature and dye concentration were discussed. Excellent dyeing behavior, levelling properties and good fastness on wool/acrylic blended fabric were obtained. What’ more, excellent anti-ultraviolet and antibacterial properties were obtained for textiles with these dyes. The application of these dyes with large molar extinction coefficients presents a wide range of possibilities for the further development of cleaner production and eco-friendly dyeing, even functional textiles.

## 1. Introduction

The application of blended fabrics greatly confers the complementary properties of fibers and reduces processing costs by combining the advantages of multiple fibers in textile goods. In recent years, the great market demand for wool/acrylic blended fabrics has remained a great impetus in knitwear because of its unique hand feeling, excellent wearing comfort, soft texture, and warmth retention [1,2]. Generally, wool/acrylic blended fabrics are carried out by the two-bath dyeing method. Compared with a conventional two-bath process [3], the one-bath dyeing method is widely applied to wool/acrylic blended fabric dyeing in the aspects of production efficiency improvement and energy saving [4]. However, large amounts of surfactants are used to avoid the reaction between cationic dyes for acrylic and anionic dyes for wool in one-bath dyeing, which has a serious effect on the environment [5]. As we all know, textile wastewater is one of the most important pollution sources in the textile industry, causing serious damage to the ecosystem and lives [6,7]. Hence, the development of dyes applied for the one-bath processing of wool/acrylic fabrics will become an active research area in printing and dyeing as it reduces the effluent and improves the dyeing effectiveness.

**Figure 1:**
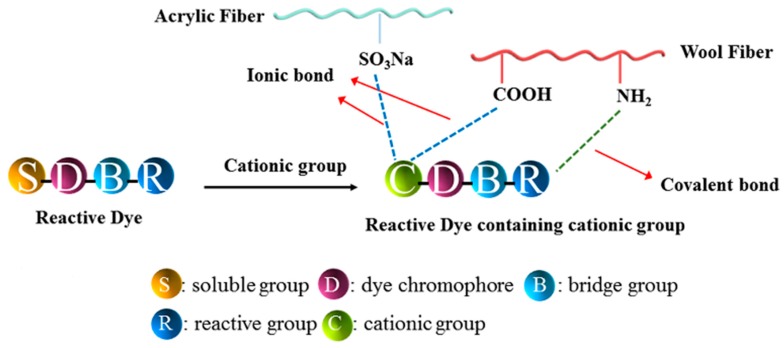
The mechanism between reactive dye with blended fabric.

In our previous work, it has proven promising to use reactive dyes containing cationic groups for the one-bath dyeing of wool/acrylic blended fabrics [8,9,10,11,12]. The reactive group in the molecular structure of dye reacts with the wool fiber to form the covalent bond and the cationic group combines with acrylic fiber via the ionic bond, realizing the one-bath dyeing of wool/acrylic blended fabrics (shown in Figure 1) [13]. To date, a series of reactive dyes containing cationic groups reported with red and yellow based on aniline-based and anthraquinone as chromophores has been described [14,15,16,17]. However, most reactive dyes have shown little substantivity for wool and acrylic fiber at the same time and exhibited some shortages in terms of color-deepening, color fastness, and brightness of shade. Therefore, researchers are still looking for brighter, more effective, and amenable colorants for this family with the aim of improving the dye-uptake and the tinctorial strength [18].

Heterocyclic dyes are well-known compounds that have larger tinctorial strength and brighter color than azo dyes based on aniline-based derivative [19,20,21], and have attracted great interest among dye chemists in the modern textile chemistry in recent years [22,23]. Commercial heterocyclic dyes have been largely classified into four major classes: (benzo) thiazole dyes, (benzo) isothiazole dyes [24,25], thiadiazole dyes [26], and thiophene dyes [27]. Due to extensive delocalized π-systems, these dyes showed a significant bathochromic effect in the UV-visible absorption spectrum [28]. Green, red, blue and dark tones have been displayed on the fabrics. In addition, much work so far has focused on the effect of the aromatic rings with the substituent groups [29]. Meanwhile, the development of dyed fabrics with many functions, such as anti-ultraviolet performance [30], antibacterial activities [31,32], and sensing applications [33,34], have increased rapidly. The development of multifunctional dye has also proven promising. To the best of our knowledge, only a few researches have been reported about the one-bath dyeing of wool/acrylic blended fabrics with reactive dyes containing cationic groups based on heterocyclic chromophores. The application of these reactive dyes based on benzothiazole derivative chromophores not only provides a brighter color, but also reduces the usage of dyes to achieve same color depth.

**Figure 2:**
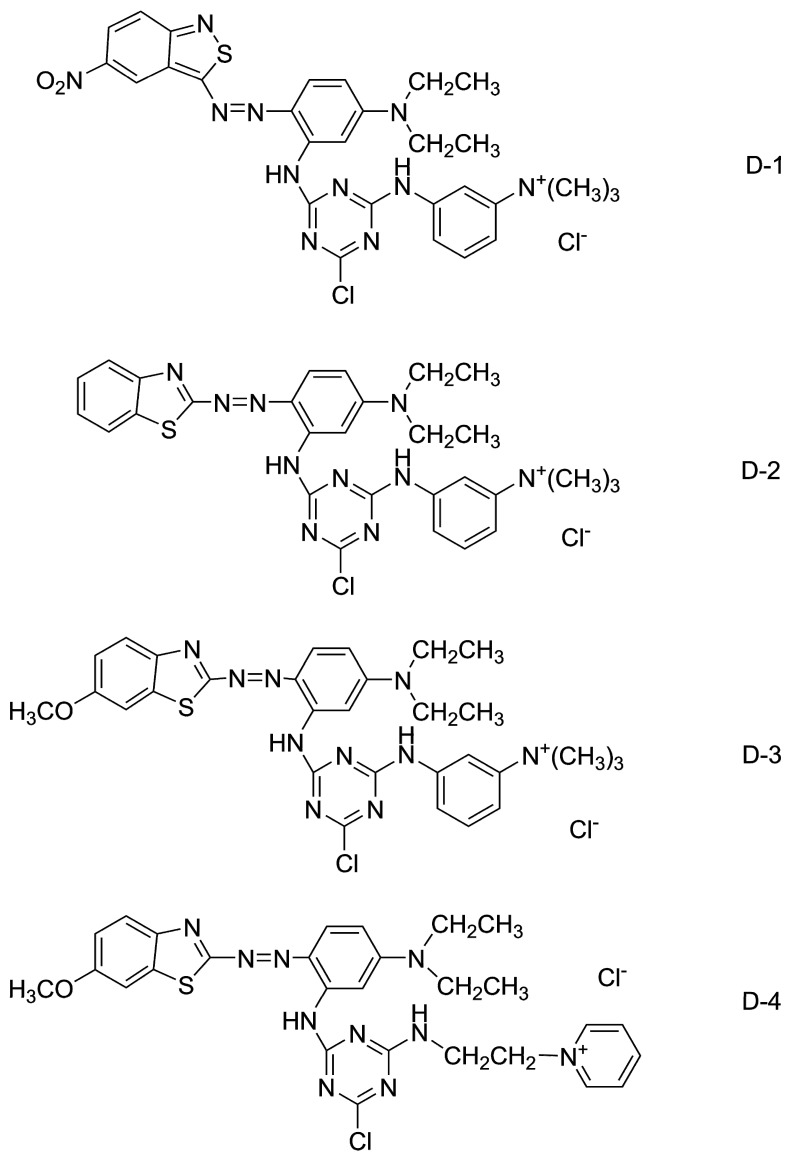
Structures of the synthesized heterocyclic reactive dyes.

In the present paper, four reactive dyes containing cationic groups based on azo benzothiazole derivative (shown in Figure 2) as a diazo component have been designed. They had a wide range of color shades with a very better depth and darker color compared with other reactive dyes reported in the literature [18,35]. In order to reveal the structure–spectra relationship between these dyes, different substituted groups have been introduced into molecule structures, and the method of density function theory (DFT) was recommended. The dyeing behaviors of these dyes were evaluated by determining the color strength (K/S) value on wool/acrylic fabric in order to further elucidate the influence of dyeing conditions, including pH, temperature, and time. The levelling properties and color fastness such as washing, rubbing, and light fastness were measured. What’s more, the anti-ultraviolet performance and antibacterial activities against *S. aureus* of dyed textiles were investigated.

## 2. Experimental

### 2.1. Materials and Instrumentation

Cyanuric chloride, 2-amino-6-methoxybenzothiazole and 2-aminobenzothiazole were supplied by the Tokyo Chemical Industry Co., Ltd. (TCI, Shanghai, China) and used without any further purification. Further, 3-amino-5-nitrobenzoisothiazole, 3-(*N, N*-diethylamino) acetanilide were received from Wanfeng Chemical Engineering Co., Ltd (Zhejiang, China). All other reagents were obtained from Shanghai Chemical Reagent Co., Ltd. (Shanghai, China).

Wool (265.1 g/m^2^), acrylic (150 g/m^2^) and 50/50 wool/acrylic blended fabric (150 g/m^2^) were provided by Donghua University Textile Institute Training Center (Shanghai, China). The fabrics were pretreated with 2 g/L nonionic detergent at 60 ℃ for 30 min before being rinsed and dried.

Ultraviolet-visible (UV-Vis) absorption spectra of these dyes, dissolved in dimethylformamide solution, were recorded with the U-3310 Spectrophotometer (Hitachi Limited, Tokyo, Japan) using a quartz glass cell at the room temperature. The color strength (K/S) and colorimetric data of the dyed fabrics were determined by Datacolor SF 600X spectrophotometer (Datacolor, Lucerne, Switzerland). The topography of different fibers was observed at the optical microscope. A 3D microscope graph of fabrics was obtained by Super Depth of Field 3D Microscope VHX-6000 (Keyence, Osaka, Japan). Ultraviolet protection factor (UPF) and T_UVA_% were measured by Labsphere UV 2000F (Labsphere, North Sutton, NH, USA).

### 2.2. Preparation

These four novel dyes were provided by our research group. The cationic quaternary ammonium salts including *N*-(2-aminoethyl) pyridinium chloride salt and m-aminophenyltrimethylammonium salt were synthesized according to the reported researches [16,36,37,38]. The final products were synthesized by classical condensation and diazotization-coupling reaction according to the literature [12]. Methanol and dichloromethane (4:1 by volume) as the eluent system for chromatographic column was used to purified. All the dyes were prepared successfully and investigated by FT-IR, ^1^H-NMR, and EA. The synthetic process and results can be found in supplementary materials.

### 2.3. Dyeing and Measure Methods

#### 2.3.1. One-Bath One-Step Procedure

**Figure 3:**
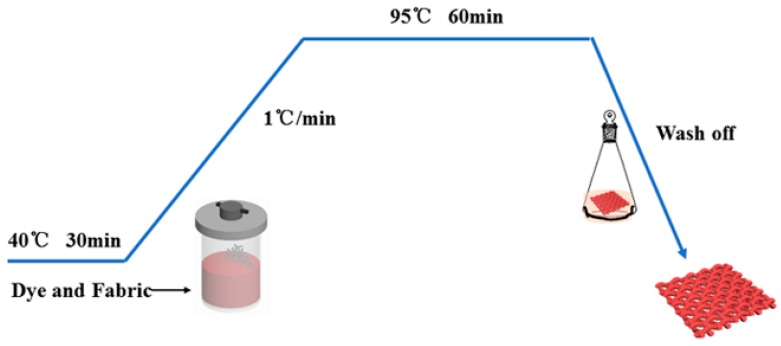
Dyeing profile of wool/acrylic blended fabric using heterocyclic dyes.

All dyeing of three kinds of fabrics were carried out using an infrared laboratory dyeing machine (PYROTEC-2001, Roaches International, UK) using four heterocyclic reactive dyes with a liquor of 40:1 at different shade of 1~5% (o. w. f). These dyes were applied to wool/acrylic blended fabric using the one-bath dyeing method as described in Figure 3. After dyeing, the dyed samples were soaped for 10 min at 98 °C in a soap solution of containing 2 g/L soap flakes.

#### 2.3.2. Dye Exhaustion

The absorbance of the dye solution before and after dyeing was carried out by UV-Vis spectrophotometry at λ_max_. The percentage of exhaustion (E%) was calculated using Equation (1):

(1) E%=(A0−A1)A0×100

where A_0_ and A_1_ are the absorbance of the dye solution before and after the dyeing process, respectively.

#### 2.3.3. The Color Strength (K/S) on the Dyed Fabrics

In the spectrum visible region, 380–720 nm, the K/S values and color parameters (L *, a *, b *) of the dyed fabric were measured using Datacolor SF 600X spectrophotometer. In order to obtain the correct K/S, five separate points of each tested dyed fabric were selected. The color yield of dyed fabrics was calculated using the Kubelka-Munk equation (Equation (2)).

(2) KS=(1−R)22R

where *R* is the reflectance of the dyed substrate at λ_max_, *K* is the absorption coefficient, and *S* is the scattering coefficient.

#### 2.3.4. Fabric Strength Test

Considering that knitted fabrics were used for dyeing, fabric strength was obtained by bursting strength according to GB/T 19976–2005. The bursting strength of textiles was determined by the steel ball method. The maximum bursting strength of this sample was recorded at a constant rate of extension testing machine (CRE, HD026N-200, Multifunctional electronic fabric strength tester, Nantong Hongda Experiment Instruments CO., LTD, Nantong, China) when the sample was broken. Each kind of fabric was measured at least five times to obtain the mean bursting strength.

#### 2.3.5. Levelling Test

Levelling test refers to the uniformity of the color of the fabric after dyeing. Five separate locations on the surface of the dyed fabric were chosen and assessed by Datacolor spectrophotometry [39,40]. Then, the mean color differences value (∆E) between the five locations were calculated using Equation (3). Under normal circumstances, ∆E < 1 means that the color difference is small, and the dyeing uniformity is good.

(3) $$∆E={\left[{\left(∆L\right)}^{2}+{\left(∆a\right)}^{2}+{\left(∆b\right)}^{2}\right]}^{1_{2}}$$

where ∆L, ∆a, and ∆b are the differences in the color parameters.

#### 2.3.6. Fastness Testing

Fastness testing for the dyed fabrics was performed according to international standards. Fastness to washing was assessed according to ISO 105-C03(2010). Fastness to the light was assessed using ISO 105-B02(2013). Fastness to rubbing was assessed following ISO 105-X12(2001).

#### 2.3.7. Anti-Ultraviolet Testing

Anti-ultraviolet testing for the dyed fabric was performed according to AATCC 183: 2014. The UPF and T_UVA_% of fabrics were measured by Labsphere UV-2000F.

#### 2.3.8. Antibacterial Testing

*Staphylococcus aureus* (*S. aureus*) was engaged to perform the antibacterial testing of the dyed fabric according to AATCC100-2009, and the antibacterial rates were calculated by using Equation (4).

(4) R%=(A−B)A×100

where R% is the percentage reduction of the bacterium, while A and B are the quantities of bacterial colonies of fabrics before and after the dyeing process, respectively.

## 3. Results and Discussion

### 3.1. Spectral Characterization

**Figure 4:**
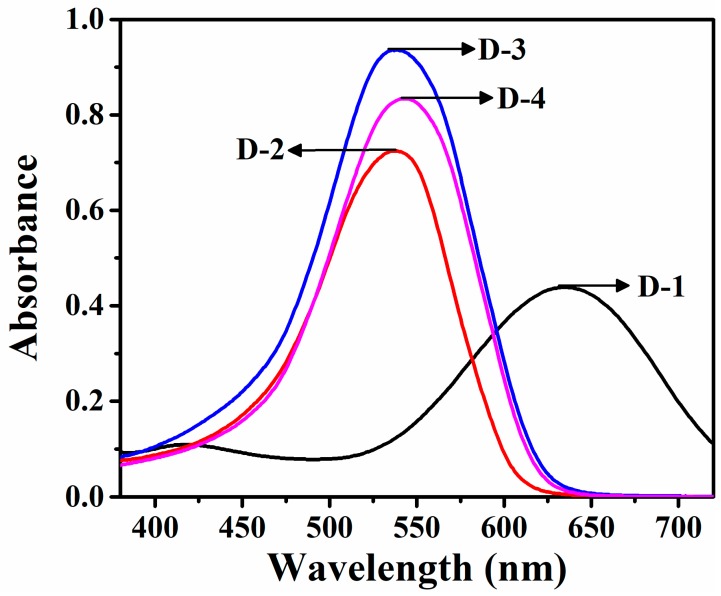
UV-Vis absorption spectra for synthetic dyes in DMF solution (0.012 g/L).

In order to investigate the relationship between spectral properties and the dye structures, the reactive dyes bearing different substituted groups were designed. The maximum absorption wavelengths of the four reactive dyes containing cationic groups with the same concentration (0.012 g/L) were measured in DMF solution. The strong absorption bands were observed in Figure 4 and results of the spectral data were listed in Table 1. The λ_max_ of them were 633.0 nm with greenish blue shade for D-1, 536.0, 535.0, and 542.0 nm with fuchsia shades for D-2, D-3, and D-4 in the visible region, respectively. It is clear to us that π-π* transition would be facilitated with the increase of the delocalized π-conjugated system [41]. The molar absorption coefficients of these dyes were brilliant at the range of 26,634 to 55,247 L·mol^−1^·cm^−1^, which was caused by the π-π* transition between the aromatic rings and the azo units.

With the common coupling components, the color and depth of hues of these dyes were influenced by the electron effect of the substituent at the diazo components. Due to the introduction of -NO_2_ substituent group as the electron-withdrawing group, the maximum absorption wavelength of D-1, is found to demonstrate a remarkable bathochromic shift. The dye was obtained by replacing diazo component of D-1 structure with 2-amino-6-nitrobenzothiazole, which still had more bathochromic shift than D-2, but its λ_max_ was shorter than that of D-1. It is known that the slight hypsochromic shift effect was usually caused by the introduction of electron-attracting groups into the “second leg” attached to the halotriazine [16]. D-3, m-aminophenyltrimethylammonium as a substituent group is more hypsochromic than D-4, *N*-(2-aminoethyl) pyridinium as substituent. As observed in Table 1, the maximum absorption of dyes did not shift significantly, indicating that they did not have a strong solvent dependence. Meanwhile, the higher the polarity of the solvent, the further λ_max_ was shifted towards a longer wavelength (bathochromism), which can be attributed to dye–solvent interactions by the dipolar effect [42] and intermolecular hydrogen bonding forces [43].

Further, the optical band gap $E_{g}^{opt}$ was determined from the onset absorption edge λ_onset_ at higher wavelengths according to Equation (5) [44]. This equation means that the greater the E, the further λ will toward hypsochromic shift. The electronic transitions cause the dye shade and are related to stimulate transition energy [45]. The optical band gap of these dyes can be found in the Table 1.

(5) Egopt=hcλonsetabs=1240/λonsetabs

**Figure 5:**
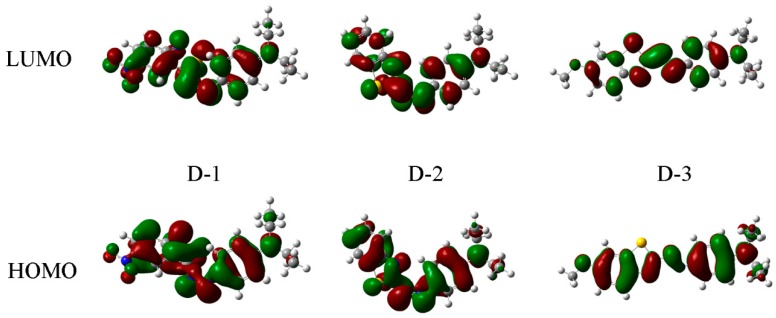
The HOMOs and LUMOs for dyes’ chromophores with DFT/B3LYP 6–31 G (d) basis.

In addition, in order to disclose the structure–spectra relation between these dyes, the DFT/B3LYP method combined with the 6–31 G (d) basis set was carried out with the Gaussian 09. The calculated energy gaps for chromophores in these dyes were 2.14, 3.11, and 2.84 eV respectively, which were in agreement with their UV-vis absorption spectra. A crude view of the processes can be provided by HOMO-LUMO plots [46]. As can be seen in Figure 5, the subtle changes for molecular orbitals including the HOMO and LUMO of these chromophores were observed due to the influences of different substituent groups attached to the heterocyclic ring, and the electron cloud density of D-3 was concentrated. As can been seen with the molecular orbital plots of HOMO and LUMO in different solvents, the electron density between HOMO and LUMO were very similar, as evident in Figure 5. It can be observed that these dyes showed a weak solvent dependency.

### 3.2. Dyeing Properties of the Four Reactive Dyes

In the dyeing process, the dye-uptake is a major factor that should be taken into consideration. All kinds of fabrics were dyed by using an exhaust dyeing process. The types of cationic groups and chromophores presented in the structures of these dyes have a remarkable effect on dyeing performance [30]. As illustrated in Figure 1, on the one hand, covalent bonds could be generated by reactive groups with the amino groups of wool fabric by nucleophilic substitution reaction, on the other hand, cationic group could combined with the carboxyl group of wool fabric and sulfonic acid group of acrylic fabric via ionic bonds.

Table 2 summarizes the percentage dye exhaustion of 1.0% (o. w. f) dyeing range. It was found that high exhaustion of D-1-D-4 could reach over 97% due to the lower solubility and good affinity for fibers. Moreover, the exhaustion on acrylic fiber of dye 4 was a little lower than that of dye 3. This implies that it’s easier for m-aminophenyltrimethylammonium as a quaternary ammonium salt group to form ionic bonds with acrylic fiber in contrast to the *N*-(2-aminoethyl) pyridinium salt group.

**Figure 6:**
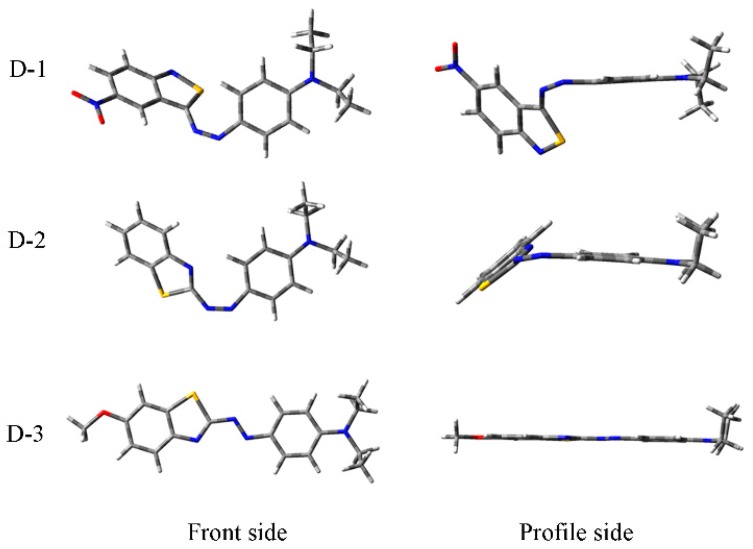
Optimized structure for the chromophore in reactive dyes.

Optimized structures for the chromophore are shown in Figure 6. The results show that, for D-3 and D-4, the dihedral angle between the phenyl and the benzothiazole rings is calculated to be 179.81°. They exhibited good planarity for the chromophore. The dye molecules with higher planarity can be easily immobilized to the surface of the fiber in a large area [47]. This also further explained the phenomenon that the exhaustion of dyes 3 and 4 were a little higher than those of dyes 1 and 2.

It is worthwhile to mention that the exhaustion of these dyes on acrylic fabric were higher than those on wool fabrics, while the K/S of dyed acrylic fabric was lower than those on wool fabrics. This implies that the color yield relates to differences of dye-uptake, dye distribution on fiber, the molecular structures of the dye, and the refractive index of fiber.

### 3.3. Effect of pH Value

**Figure 7:**
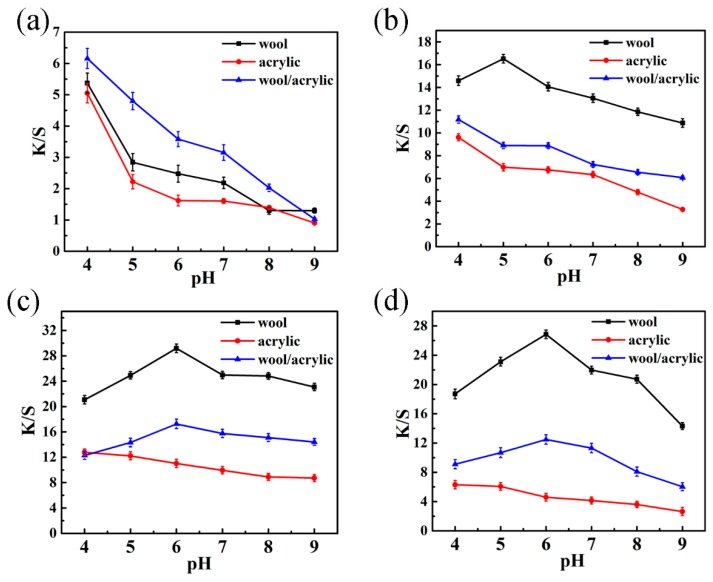
Effect of pH on K/S for heterocyclic reactive dyes. (**a**) Dye 1; (**b**) Dye 2; (**c**) Dye 3; (**d**) Dye 4.

The pH value is an important factor for different kinds of fabrics dyeing. With the exception of D-1, it can be seen from Figure 7 that the K/S of these dyes on wool fabric dyeing slightly increased with the increase of the pH value, while it decreased when the pH value exceeded 6, which is related to the isoelectric point of wool fabrics (4.2–4.8) and the hydrolysis of dyes. When the pH value of the dyebath exceeded its isoelectric point of wool, a negative charge on the surface of the wool could form an electrostatic attraction with cationic groups of reactive dye. At the same time, the number of free amino groups on the wool was increased, which is favorable for forming a covalent bond with reactive groups.

As displayed in Figure 7, pH 4 was the optimum value for acrylic fabric dyeing. It is well known that cationic dyes were applied to the acrylic fiber dyeing at pH 4–5. The color yield decreased due to the hydrolysis of the acrylic fiber and the dye under high pH value dyeing conditions [48]. Overall, as can be seen from Figure 7, for D-1 and D-2, the color yields on the wool/acrylic were maximized at pH 4 of the dyebath, but the maximum color yield for D-3 and D-4 were at pH 6.

### 3.4. Effect of Temperature

**Figure 8:**
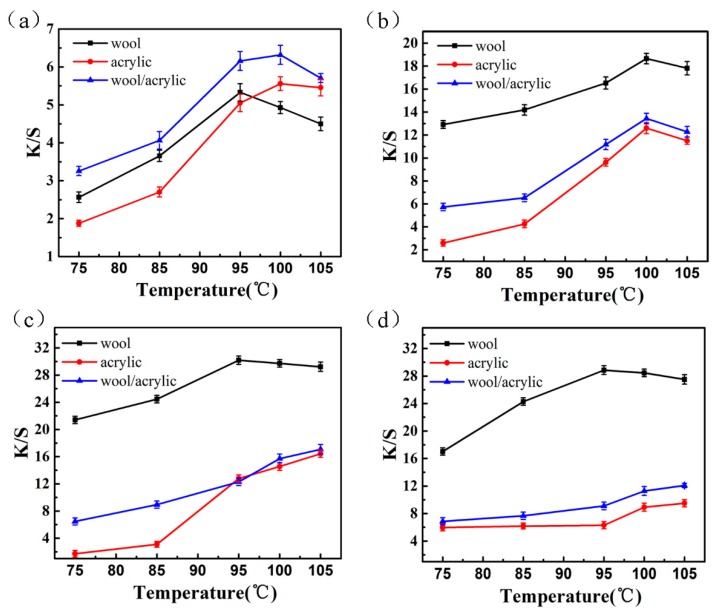
Effect of temperature on K/S for heterocyclic reactive dyes. (**a**) Dye 1; (**b**) Dye 2; (**c**) Dye 3; (**d**) Dye 4.

It can be seen in Figure 8 that the low K/S values of dyes was observed below 95 °C for acrylic fiber dyeing. However, the K/S increased significantly when the dyeing temperature exceeded the glass transition temperature of the acrylic fiber (80~90 °C), at which temperature the molecular segment movement was intensified, and the dye molecules were more likely to diffuse into the fiber interior.

**Figure 9:**
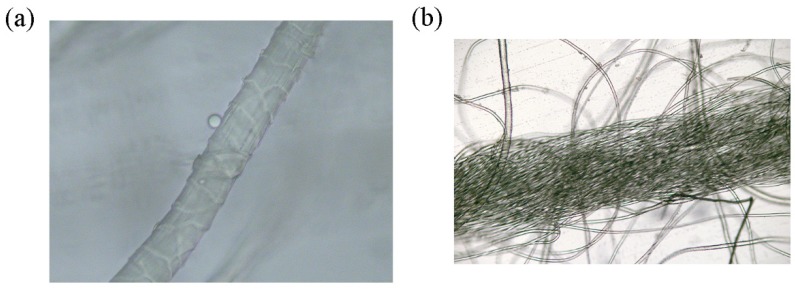

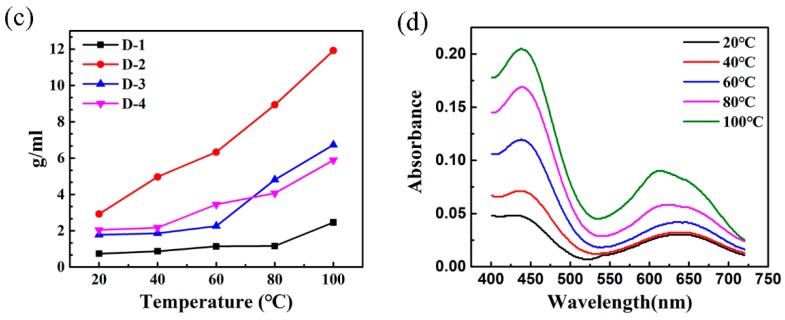
(**a**) The topography of wool fiber; (**b**) The topography of wool/acrylic blended fiber; (**c**) The solubility of these dyes under the different temperatures; (**d**) The absorbance change of D-1 with temperature.

An increase in the K/S values of dyed wool fabrics was observed with the increase of temperature, which attributed to the dense scale layers on the wool surface. The topography of wool fiber was observed by the optical microscope (Figure 9a). On the one hand, as the temperature ascended, the scale layer on the wool surface opened, and the resistance between fibers and dyes became smaller. On the other hand, as can be seen in Figure 9c, the solubility of these dyes gradually increased.

Additionally, the K/S value tended to be smooth and even declined when the temperature exceeded 100 °C owing to the hydrolysis of the dye. As shown in Figure 9d, the heterocyclic structure of D-1 was especially unstable under high temperature. The results indicate that 95 °C is the suitable temperature as a one-bath dyeing contact temperature on the wool/acrylic blended fabric.

### 3.5. Dyeing Heat-Rate Curve

**Figure 10:**
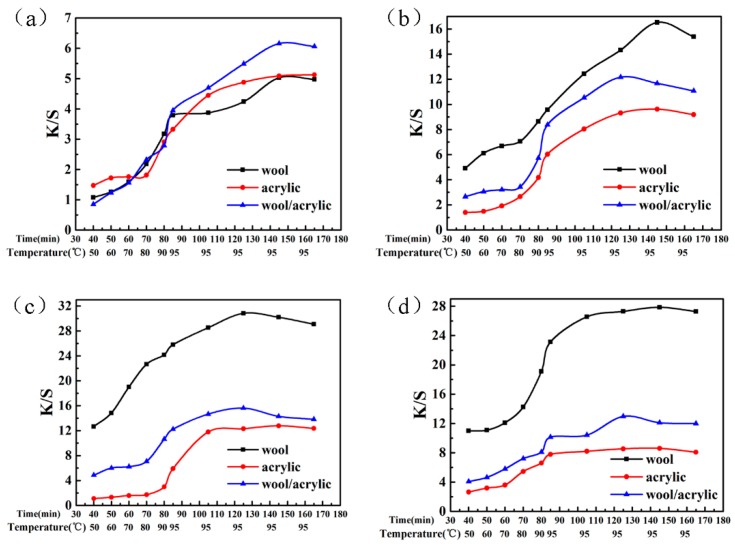
The dyeing heat-rate curve of the heterocyclic reactive dyes. (**a**) Dye 1; (**b**) Dye 2; (**c**) Dye 3; (**d**) Dye 4.

Dyeing heat-curves of these dyes are described in Figure 10. It is clear that increasing the dyeing temperature and time has a pronounced effect on the K/S value of the dyed fabric. This phenomenon is also attributed to the fiber swelling and dye diffusion. What’s more, the dyeing heat-rate curves of these dyes were provided by comparing the K/S value between three types of fabrics.

As shown in Figure 10, the dye-uptake on wool increased gradually with dyeing temperature and time, but the dye-uptake on acrylic fiber increased sharply at above 90 °C, which can be attributed to the rapid motion of the chain segments above the glass transition temperature. What’ more, the dyeing heat-rate curve and the K/S value of these dyes on blended fabric were similar to those on acrylic fabric owing to the order of dyeing different fibers [13].

### 3.6. Build-Up and Homochromaticity of Different Dyed Fabrics

**Figure 11:**
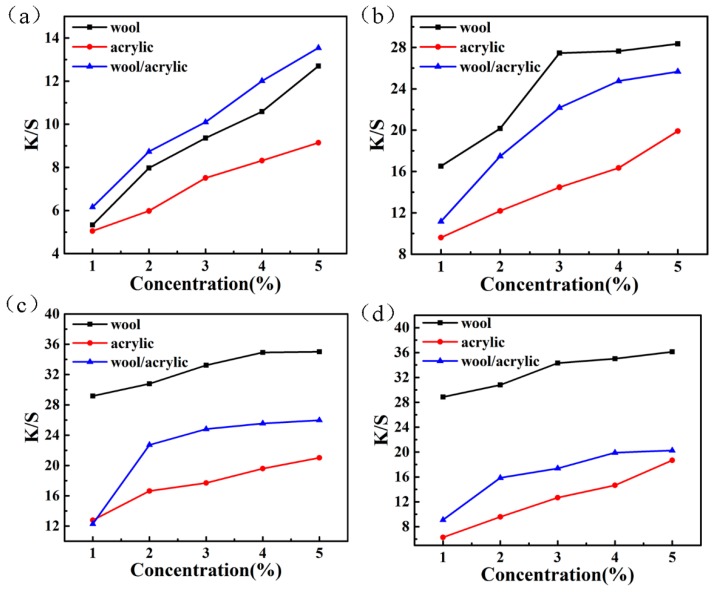
Build-up property of the heterocyclic reactive dyes. (**a**) Dye 1; (**b**) Dye 2; (**c**) Dye 3; (**d**) Dye 4.

The build-up property of three kinds of dyed fabrics is significant in terms of dye utilization and production cost [49], as shown in Figure 11. When the dye concentration reaches to 4% (o. w. f), the K/S value increases slowly, indicating that the dyeing tends to be saturated. The K/S value of the wool/acrylic blended fabric locates between the single-component fiber with the same concentration. This can be explained by the fact that the synergistic effect of the two fibers in the blended fabric during the exhaustion of the dyes.

The union dyeing property (k) is an important index to determine the color match between dyed acrylic and wool fabric, which were calculated using Equation (4).

(6) k=(KS)wool(Ks)acrylic

where (K/S)_wool_ and (K/S)_acrylic_ are the K/S values of dyed wool fabric and acrylic fabric, respectively.

The parameters (refers to L *, a *, and b * values) of the dyed fabrics at 1.0% (o. w. f) dye concentration are shown in Table 3. The hue variations were observed by combining all parameters. Generally, the union dyeing properties were good in the case where k is close to 1 [50]. Meanwhile, the low color differences between acrylic and wool fiber can meet the requirements in the practical application. In general, D-1 has more outstanding union dyeing properties than the other dyes.

### 3.7. Fabric Strength Test

Three fabrics were dyed with a liquor of 40:1 at 95 °C, 60 min, and 1.0% (o. w. f) dye concentration. The fabric strength of three fabrics was shown in Table 4. The results showed that the fabric strength of dyed fabrics was slightly lower than those of untreated fabric, but there was no obvious difference. The decrease of fabric strength may be caused by temperature. In order to accelerate the diffusion of dyes and motion of the chain segments, a higher temperature was used during the process of dyeing. For this dyeing process, the damage of fiber was caused by higher temperature, reducing the fabric strength of dyed fabrics. However, compared with untreated fabrics, the dyeing condition had little influence on the fabric strength.

### 3.8. Levelling Properties

**Figure 12:**
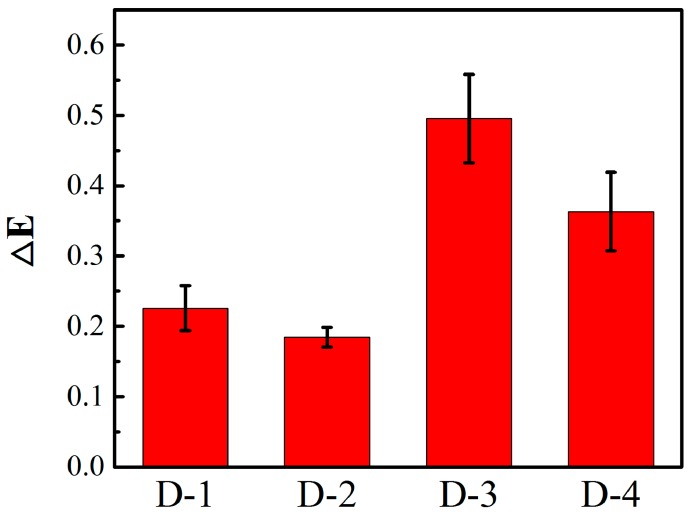
Levelling properties of wool/acrylic blended fabrics with heterocyclic dyes.

**Figure 13:**
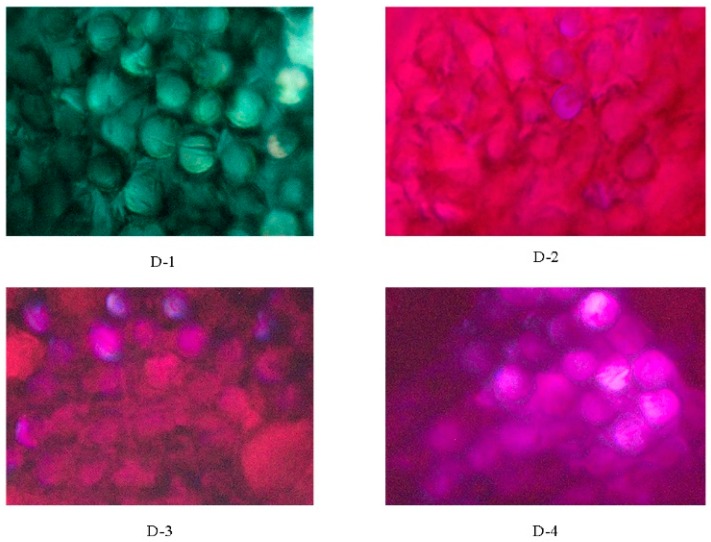
3D microscope graph of wool/acrylic blended fabrics with heterocyclic dyes.

The dyed wool/acrylic blended fabrics at 1.0% (o. w. f) dye concentration were measured by a Datacolor spectrophotometer to obtain the mean color differences (ΔE). As shown in Figure 12, these dyed fabrics had low color differences and good levelling properties. The 3D microscope graph in Figure 13 shows that each fiber was evenly dyed and viewed from the cross-section of the fiber. The two kinds of fiber interweaved and cooperated with each other and the dyes were transferred among fibers, achieving the dyeing equilibrium. The levelling properties of D-1 and D-2 were better than D-3 and D-4, which was ascribed to its similar color strengths.

### 3.9. Fastness Properties of the Dyed Fabrics

The color fastness properties of the dyed blended fabric with four dyes bearing an azo benzothiazole derivate at 1.0% (o. w. f) dye concentration were summarized in Table 5. The results showed that the dry rubbing was good, but wet rubbing fastness was poor, which can be explained by the existence of dye oligomer. Further, D-4 containing *N*-(2-aminoethyl) pyridinium as cationic group had better light fastness than the other dyes, but they were grades 4–5 due to the benzothiazole structure.

### 3.10. Anti-Ultraviolet Properties of Dyed Fabric

**Figure 14:**
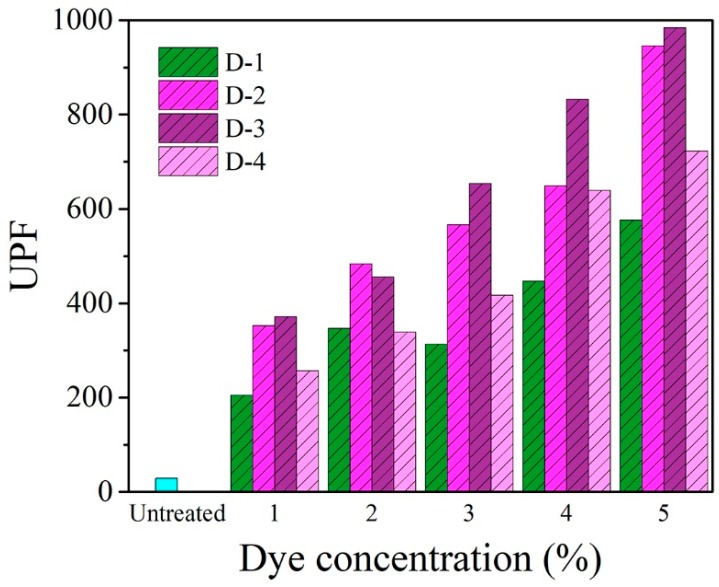
Anti-ultraviolet properties of wool/acrylic blended fabrics with these dyes.

The effects of dyes on the anti-ultraviolet properties of dyed fabric were investigated and shown in Figure 14. According to the assessment standard of the anti-UV performance of textile, the textiles whose UPF is better than 40 and T_UVA_% is less than 5% can be called the UV-protection products. Compared with untreated fabric, the UPF of dyed fabrics has been remarkably improved and the T_UVA_% of these four dyes was less than 0.5%. The data of UPF and T_UVA_% demonstrate that they exhibited excellent anti-ultraviolet properties. Further, results showed that the UV resistance of dyed wool/acrylic blended fabrics improved with increasing the dosage of four novel heterocyclic dyes owing to the increase of dye adsorption capacity.

### 3.11. Antibacterial Properties of Dyed Fabric

According to the AATCC-100 antibacterial test method, the antibacterial rate of dyed wool/acrylic blended fabrics against *Staphylococcus aureus* was evaluated at 5.0% (o. w. f) dye concentration. As can been seen in Table 6, the antibacterial rates of dyed wool/acrylic blend were around 90%. Quaternary ammonium groups in the molecular structure played a major role in the antibacterial activities, which caused the damage of the cell membrane and protein activity [51,52,53]. At the same time, the free electrons on the surface of N and S atoms in the structures had an auxiliary effect on the antibacterial activities. Such a phenomenon indicates that the dyed fabric possessed effective antibacterial activities. Further, measurements of the effect on hydrocarbon chain length and washing durability of antimicrobial functions are ongoing in our laboratory.

## 4. Conclusions

Four novel reactive dyes containing cationic groups based on an azo benzothiazole derivative chromophores were designed and applied to wool/acrylic blended fabric by the one-union dyeing. These results demonstrate that these reactive dyes based on heterocyclic chromophores show beautiful color with high molar extinction coefficients. Moreover, these dyes exhibited excellent dyeing properties and fastness on wool/acrylic blended fabric. For wool/acrylic blended fabric dyed with D-1 and D-2, the optimum dyeing conditions were determined at pH 4, 95 °C for 60 min. In contrast, pH 6 is suitable for D-3 and D-4 dyes. Further, these dyes possessed excellent anti-ultraviolet properties and antibacterial activities. The present popularity of the reactive dyes containing cationic groups can be ascribed to their application of environmentally friendly functional finishing. This work will open the door for many dye chemists to improve the dye-uptake and color fastness and to enrich the applications of dyes on wool/acrylic textiles.

## Figures and Tables

**Table 1:** Spectroscopic properties of the azo heterocycle reactive dyes.

Dye	λ_DMF_ (nm)	λ_Methanol_ (nm)	λ_Isopropanol_ (nm)	ε (L mol^−1^ cm^−1^)	Color	λ_onset_ (nm)	$E_{g}^{opt}$ (ev)
D-1	633.0	623.0	621.0	26634	greenish blue	712.0	1.74
D-2	536.0	535.0	532.0	34435	fuchsia	614.0	2.02
D-3	535.0	534.0	532.0	55247	fuchsia	626.0	1.98
D-4	542.0	539.0	536.0	43379	fuchsia	624.0	1.99

**Table 2:** Exhaustion and K/S value of synthesized dyes.

		Wool Fabric	Acrylic Fabric	Wool/Acrylic Blended Fabric
D-1	E%	97.68%	99.94%	97.85%
	K/S	5.33	5.05	6.16
D-2	E%	99.86%	99.75%	99.44%
	K/S	16.52	9.61	11.18
D-3	E%	99.91%	99.43%	99.97%
	K/S	29.18	12.77	12.29
D-4	E%	99.89%	98.71%	99.91%
	K/S	28.85	6.30	9.10

**Table 3:** The union dyeing performance of wool and acrylic fabric.

Dyes	Fabrics	L *	a *	b *	K/S	k
D-1	wool	39.40	−16.23	−7.43	5.33	1.06
	acrylic	41.49	−15.82	−7.61	5.05	
D-2	wool	28.24	38.15	−0.97	16.52	1.72
	acrylic	32.41	34.80	−6.62	9.61	
D-3	wool	20.71	24.52	−3.82	29.18	2.29
	acrylic	28.64	31.32	−10.21	12.77	
D-4	wool	22.47	32.29	−9.20	28.85	4.58
	acrylic	28.48	19.64	−4.54	6.30	

**Table 4:** The fabric strength of three fabrics before and after dyeing.

	Wool Fabric	Acrylic Fabric	Wool/Acrylic Fabric
Untreated fabric	219.1 N	331.8 N	379.3 N
Dyed fabric with D-1	213.5 N	325.3 N	373.1 N
Dyed fabrics with D-2	212.8 N	324.6 N	372.2 N
Dyed fabrics with D-3	212.5 N	324.7 N	372.5 N
Dyed fabrics with D-4	214.6 N	325.5 N	373.4 N

**Table 5:** Fastness of dyed wool/acrylic blended fabrics at 1.0% (o. w. f) dye concentration ^a^.

Samples	Digital Pictures for Blended Fabric (1%owf)	Rubbing Fastness		Washing Fastness			Light Fastness
		Dry	Wet	SC	SW	SA	
D-1	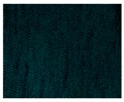	5	4	4	4	4–5	4
D-2	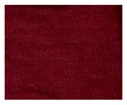	5	4	4	4	4–5	4–5
D-3	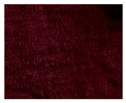	4–5	3–4	4	4	4–5	4–5
D-4	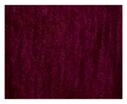	4–5	4	4–5	4–5	4–5	4–5

^a^ SC = staining on cotton; SW = staining on wool; SA = staining on acrylic.

**Table 6:** The antibacterial rates of dyed wool/acrylic blended fabrics.

Types	Undyed Fabric	D-1	D-2	D-3	D-4
Antibacterial rate (%)	0	88.09	91.23	94.50	90.46

## Supplementary Materials

The following are available online at https://www.mdpi.com/2073-4360/12/2/285/s1.

## References

[1] Zhang Y., Zhang W., Cheng Y., Zhang R., Qin C., Chen G. (2016). Preparing Fluorescent Wool/Acrylic Blends with a Hemicyanine Reactive Cationic Dye. Fibers Polym..

[2] El-Shishtawy R.M., El-Zawahry M.M., Ahmed N.S.E. (2011). One-bath union dyeing of a modified wool/acrylic blend with acid and reactive dyes. Color. Technol..

[3] Lee J.J., Han N.K., Lee W.J., Choi J.H., Kim J.P. (2003). One-bath dyeing of a polyester/cotton blend with reactive disperse dyes from 2-hydroxypyrid-6-one derivatives. Color. Technol..

[4] Sadeghi-Kiakhani M., Gharanjig K., Arami M. (2014). Study on dyeing and fastness properties of wool-polyester blend fabrics using novel mono azo-naphthalimide dyes. J. Text. Inst..

[5] Wang J., Zhang Y., Dou H., Pei L. (2018). Influence of Ethylene Oxide Content in Nonionic Surfactant to the Hydrolysis of Reactive Dye in Silicone Non-Aqueous Dyeing System. Polymers.

[6] Lin J., Lin F., Chen X., Ye W., Li X., Zeng H., Van der Bruggen B. (2019). Sustainable Management of Textile Wastewater: A Hybrid Tight Ultrafiltration/Bipolar-Membrane Electrodialysis Process for Resource Recovery and Zero Liquid Discharge. Ind. Eng. Chem. Res..

[7] Fleischmann C., Lievenbrück M., Ritter H. (2015). Polymers and Dyes: Developments and Applications. Polymers.

[8] Xiao H., Zhao T. (2018). One-Bath Union Dyeing of Wool/Acrylic Blend Fabric with Cationic Reactive Dyes Based on Azobenzene. Fibers Polym..

[9] Zhao T., Gehui W. (2010). Dyeing and Antimicrobial Properties of Antimicrobial Cationic Reactive Dye on Different Kind of Fibers. J. Donghua Univ..

[10] Xiao H., Zhao T., Li C.H., Li M.Y. (2017). Eco-friendly approaches for dyeing multiple type of fabrics with cationic reactive dyes. J. Clean Prod..

[11] Xiao H., Wang M., Zhao T. (2016). Dyeing performance of reactive cationic dyes. Dye. Finish..

[12] Xiao H., Li C., Wang P., Zhao T. (2019). A feasible approach for enhancing union dyeing of wool/acrylic blend fabrics with heterobifunctional cationic reactive dyes. Text. Res. J..

[13] Xie K.L., Hou A.Q. (2004). One-bath dyeing of wool/acrylic blends with reactive cationic dyes based on monofluorotriazine. Color. Technol..

[14] Wang G.W., Zheng C.L., Sun J. (2016). Synthesis and salt-free dyeing characteristics of cationic reactive dyes containing polyetheramine segments. Color. Technol..

[15] Javadi M.S., Mokhtari J. (2012). Synthesis and Evaluation of Technical Properties of Novel Cationic Mono-s-chloro Triazinyl (MCT) Reactive Dyes on Cotton. J. Chin. Chem. Soc..

[16] Soleimani-Gorgani A., Taylor J.A. (2008). Dyeing of nylon with reactive dyes. Part 3: Cationic reactive dyes for nylon. Dyes Pigment..

[17] Liu J., Sun G. (2008). The synthesis of novel cationic anthraquinone dyes with high potent antimicrobial activity. Dyes Pigment..

[18] Soleimani-Gorgani A., Taylor J.A. (2011). Synthesis and evaluation of a novel blue cationic reactive dye for modified nylon 6.6 ‘Tactel Coloursafe’. Color. Technol..

[19] Shah S.S., Ahmad R., Shah S.W.H., Asif K.M., Naeem K. (1998). Synthesis of cationic hemicyanine dyes and their interactions with ionic surfactants. Colloids Surf. A.

[20] Şener N., Gür M., Çavuş M.S., Zurnaci M. (2019). Synthesis, Characterization, and Theoretical Calculation of New Azo Dyes Derived from [1,5]Pyrimidinene Having Solvatochromic Properties. J. Heterocyclic. Chem..

[21] Ho Y.W., Yao W.H. (2006). Synthesis and properties of heterocyclic monoazo dyes derived from 3-cyano-4-trifluoromethyl-6-substituted-2(1H)-pyridinethiones. Dyes Pigment..

[22] Wang Y.G., Wang Y.H., Tao T., Qian H.F., Huang W. (2015). Structural and spectral comparisons between isomeric benzisothiazole and benzothiazole based aromatic heterocyclic dyes. J. Mol. Struct..

[23] Gao A., Zhang H., Hou A., Xie K. (2017). Dyeing properties of the disperse dyes containing cyano group based on benzisothiazole for polyester fabrics under alkali condition. Fibers Polym..

[24] Qian H.F., Wang Y.G., Chen X.C., Ruan W.G., Huang W. (2013). Structural and spectral characterizations of C.I. Disperse Blue 148 having a new crystalline form. Dyes Pigment..

[25] Elgazwy A. (2003). The chemistry of isothiazoles. Tetrahedron.

[26] Maradiya H.R., Patel V.S. (2002). Dyeing performance of disperse dyes based on 2-aminothiazole for cellulose triacetate and nylon fibers. Fibers Polym..

[27] Yen M. (2004). A facile syntheses and absorption characteristics of some monoazo dyes in bis-heterocyclic aromatic systemspart II: Syntheses of 4-(p-substituted) phenyl-2-(2-pyrido-5-yl and 5-pyrazolo-4-yl) azo-thiazole derivatives. Dyes Pigment..

[28] Tao T., Xu F., Chen X.C., Liu Q.Q., Huang W., You X.Z. (2012). Comparisons between azo dyes and Schiff bases having the same benzothiazole/phenol skeleton: Syntheses, crystal structures and spectroscopic properties. Dyes Pigment..

[29] Pu S., Liu W., Liu G. (2010). The photochromism of unsymmetrical diarylethene isomers with an electron-withdrawing cyano substituent. Dyes Pigment..

[30] Riva A., Algaba I., Pepio M., Prieto R. (2009). Modeling the Effects of Color on the UV Protection Provided by Cotton Woven Fabrics Dyed with Azo Dyestuffs. Ind. Eng. Chem. Res..

[31] Mohamed F.A., Abd El-Megied S.A., Bashandy M.S., Ibrahim H.M. (2018). Synthesis, application and antibacterial activity of new reactive dyes based on thiazole moiety. Pigm. Resin Technol..

[32] Silva M.G.d., Barros M.A.S.D.d., Almeida R.T.R.d., Pilau E.J., Pinto E., Soares G., Santos J.G. (2018). Cleaner production of antimicrobial and anti-UV cotton materials through dyeing with eucalyptus leaves extract. J. Clean Prod..

[33] Jeevarathinam A.S., Pai N., Huang K., Hariri A., Wang J., Bai Y., Wang L., Hancock T., Keys S., Penny W. (2019). A cellulose-based photoacoustic sensor to measure heparin concentration and activity in human blood samples. Biosens. Bioelectron..

[34] Martinez A.W., Phillips S.T., Whitesides G.M., Carrilho E. (2010). Diagnostics for the Developing World: Microfluidic Paper-Based Analytical Devices. Anal. Chem..

[35] Aktek T., Millat A.K.M.M. (2017). Salt Free Dyeing of Cotton Fiber—A Critical Review. Int. J. Text. Sci..

[36] Srikulkit K., Santifuengkul P. (2000). Salt-free dyeing of cotton cellulose with a model cationic reactive dye. J. Soc. Dyers Colour..

[37] Farouk R., Gaffer H.E. (2013). Simultaneous dyeing and antibacterial finishing for cotton cellulose using a new reactive dye. Carbohydr. Polym..

[38] Zheng C., Yuan A., Wang H., Sun J. (2012). Dyeing properties of novel electrolyte-free reactive dyes on cotton fibre. Color. Technol..

[39] Xie K., Gao A., Li M., Wang X. (2014). Printing properties of the red reactive dyes with different number sulfonate groups on cotton fabric. Carbohydr. Polym..

[40] El-Shishtawy R.M., Youssef Y.A., Ahmed N.S.E., Mousa A.A. (2007). The use of sodium edate in dyeing: II. Union dyeing of cotton/wool blend with hetero bi-functional reactive dyes. Dyes Pigment..

[41] Xu D., Li Z., Peng Y.X., Geng J., Qian H.F., Huang W. (2016). Post-modification of 2-formylthiophene based heterocyclic azo dyes. Dyes Pigment..

[42] Hamidian H., Zahedian N., Ghazanfari D., Fozooni S. (2013). Synthesis and Evaluation of Changes Induced by Solvent and Substituent in Electronic Absorption Spectra of New Azo Disperse Dyes Containig Barbiturate Ring. J. Spectrosc..

[43] Mohammadi A., Yazdanbakhsh M.R., Farahnak L. (2012). Synthesis and evaluation of changes induced by solvent and substituent in electronic absorption spectra of some azo disperse dyes. Spectrochim. Acta A.

[44] Geiger T., Kuster S., Yum J.H., Moon S.J., Nazeeruddin M.K., Grätzel M., Nüesch F. (2009). Molecular Design of Unsymmetrical Squaraine Dyes for High Efficiency Conversion of Low Energy Photons into Electrons Using TiO_2_ Nanocrystalline Films. Adv. Funct. Mater..

[45] Zhan Y.Z., Zhao X., Wang W. (2017). Synthesis and application of phthalimide disperse dyes. Fibers Polym..

[46] Brédas J.L. (2017). Organic Electronics: Does a Plot of the HOMO–LUMO Wave Functions Provide Useful Information?. Chem. Mater..

[47] Jiang H., Zhang L., Cai J., Ren J., Cui Z., Chen W. (2018). Quinoidal bithiophene as disperse dye: Substituent effect on dyeing performance. Dyes Pigment..

[48] El Gabry L.K. (2004). Effect of mineral acids on the properties of acrylic fabrics. Color. Technol..

[49] Zhang W., Yang Z.Y., Cheng X.W., Tang R.C., Qiao Y.F. (2019). Adsorption, Antibacterial and Antioxidant Properties of Tannic Acid on Silk Fiber. Polymers.

[50] Zheng J., Liu X., Rex Brady P. (2008). One-bath union dyeing of wool/polytrimethylene terephthalate blends. Color. Technol..

[51] Kim Y.H., Sun G. (2001). Durable antimicrobial finishing of nylon fabrics with acid dyes and a quaternary ammonium salt. Text. Res. J..

[52] Simoncic B., Tomsic B. (2010). Structures of Novel Antimicrobial Agents for Textiles—A Review. Text. Res. J..

[53] Wang X., Tang R., Zhang Y., Yu Z., Qi C. (2016). Preparation of a Novel Chitosan Based Biopolymer Dye and Application in Wood Dyeing. Polymers.

