# Supplementary material for: A Feasible Method Applied to One-Bath Process of Wool/Acrylic Blended Fabrics with Novel Heterocyclic Reactive Dyes and Application Properties of Dyed Textiles
Source: Polymers (Basel). 2020 Feb 1;12(2):285. doi: 10.3390/polym12020285 (PMC7077430; doi:10.3390/polym12020285)
Supplement: Supplementary file 1 [file polymers-12-00285-s001.pdf]

A feasible method applied to one-bath process of wool/acrylic blended fabrics with novel heterocyclic reactive dyes and application properties of dyed textiles

Meihui Wang [1], Xianfeng Wang [1], Chong Guo [1], Tao Zhao [1,2,*], Wenyao Li [3,*]

[1] College of Chemistry, Chemical Engineering and Biotechnology, Donghua University, Shanghai 201620, China; 2150561@mail.dhu.edu.cn (M.W.); Wxfrain1102@outlook.com (X.W.); 15971473653@163.com (C.G.)

[2] Key Laboratory of Science and Technology of Eco-Textile, Ministry of Education, Donghua University, Shanghai 201620, China;

[3] College of Material Engineering, Shanghai University of Engineering Science, Shanghai 201620, China

*Correspondence: tzhao@dhu.edu.cn (T.Z.); liwenyao314@gmail.com (W.L.); Tel.: +86-021-67792811(T.Z.)

S1: Synthesis and Characterization of dyes

The synthesis routes of condensation and diazotization-coupling reaction for the novel reactive dyes are shown in Scheme 1. The cationic quaternary ammonium salts including m-aminophenyltrimethylammonium salt and *N*-(2-aminoethyl) pyridinium chloride salt were synthesized according to the reported researches. Firstly, cyanuric chloride (22.14g, 0.12mol) was stirred for 1h in an ice-salt bath and then added dropwise to the above solutions of two kinds of quaternary ammonium salt (40mL, 0.1mol), respectively. The mixture was stirred for 3h at 0~5 °C, and after adjusting to pH 4-4.5 using the saturated $NaHCO_3$ solution. When the reaction was over, the solution of *N, N*-diethyl-1,3-benzenediamine prepared was added to the flask. The

reaction was stirred at 35~40 °C for further 5h, and the saturated $NaHCO_3$ solution was added to remain the pH at a range from 5.5 to 6. The TLC using acetone: n-hexane (1:4 by volume) was used to follow the progress of the reaction. The coupling components containing cationic groups were obtained and prepared for the next coupling reaction with diazonium salt.

Secondly, the final products were synthesized by classical coupling reactions. Freshly diazonium salt solution was added dropwise for 0.5h to the reaction mixture of the coupling component in a three-necked flask immersed in an ice bath under mechanical stirring. The pH was adjusted to 5-6 using aqueous ammonia solution and stirring was continued for 5h. The synthesized dyes based on 3-amino-5-nitrobenzoisothiazole, 2-aminobenzothiazole, 2-amino-6-methoxybenzothiazole, were called as D-1, D-2, D-3 and D-4, respectively. These crude products separated by filtered, collected and air dried. Methanol and dichloromethane (4:1 by volume) as the eluent system for chromatographic column was used to purified.

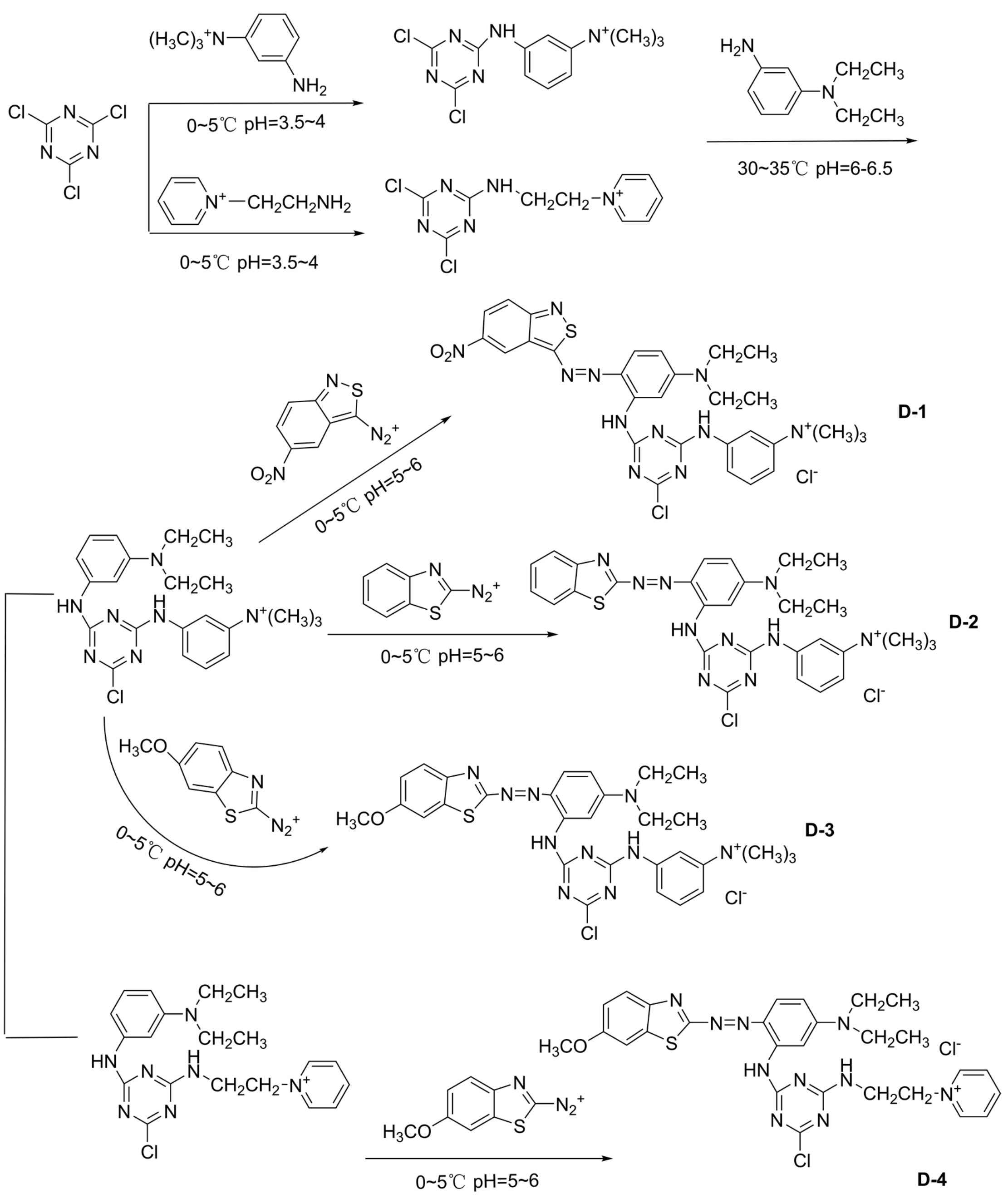

Scheme 1 Preparation routes for D-1 to D-4

Measurement:

Fourier transform infrared (FT-IR) spectra in the region of 4000-500cm^{-1} were carried out on Nexus-670 FT-IR spectrophotometer. Proton nuclear magnetic resonance (^{1}H NMR) spectra were recorded on a Bruker Advance 400 NMR spectrometer with dimethyl sulfoxide (DMSO) as the solvent and tetramethylsilane (TMS) as internal

standard. Elemental Analysis (EA) were performed on an Elmentar Vario EL III analyzer.

FT-IR, ^{1}H-NMR and EA of all the dyes are given below.

D-1, the yield was 84.91 %, FT-IR (ν cm^{-1}): 3364, 2960, 2913, 2870, 2848, 1614, 1549, 1482, 1467, 1408, 1366, 796; ^{1}H-NMR (DMSO-d$_6$, δ_H, ppm): 8.89(d, H, Ar-H), 8.15(d, 2H, Ar-H), 7.75(s, H, Ar-H), 7.69(d, 2H, Ar-H), 7.30(d, H, Ar-H), 7.11(s, H, Ar-H), 6.81 (d, H, Ar-H), 6.45(d, H, Ar-H), 3.65(s, 2H, -NH), 3.59(q, 4H, N-CH$_2$-), 1.23(s, 9H, N$^+$-CH$_3$)1.16(t, 6H, -CH$_2$-CH$_3$); Anal. Calcd. for $C_{29}H_{31}Cl_2N_{11}O_2S$: C, 52.10%; H, 4.67%; N, 23.04%. Found: C, 52.21%; H, 4.59%; N, 22.93%.

D-2, the yield was 54.82%, FT-IR (ν cm^{-1}): 3326, 2962, 2913, 2868, 2848, 1617, 1577, 1544, 1477, 1422, 1352, 791, 751; ^{1}H-NMR (DMSO-d$_6$, δ_H, ppm):7.98(d, 1H, Ar-H) , 7.83(d, 1H, Ar-H) , 7.50(t, 1H, Ar-H) , 7.42(t, 1H, Ar-H) , 7.11(t, 1H, Ar-H) , 6.84(d, 2H, Ar-H) , 6.81(d, 2H, Ar-H) , 6.43(s, 1H, Ar-H) , 6.41(d, 1H, Ar-H) , 3.63(s, 2H, -NH), 3.25(q, 4H, N-CH$_2$-), 1.23(s, 9H, N$^+$-CH$_3$), 1.12(t, 6H, C-CH$_3$); Anal. Calcd. for $C_{29}H_{32}Cl_2N_{10}S$: C, 55.86%; H, 5.17%; N, 22.46%. Found: C, 55.75%; H, 5.28%; N, 22.40%.

D-3, the yield was 72.79%, FT-IR (ν cm^{-1}): 3394, 2960, 2914, 2873, 2848, 1617, 1594, 1577, 1549, 1424, 1352, 1207, 1163, 894, 851, 791, 763; ^{1}H-NMR (DMSO-d$_6$, δ_H, ppm): 7.86(d, 2H, Ar-H), 7.81(d, H, Ar-H), 7.78(s, H, Ar-H), 7.53(d, 2H, Ar-H), 7.11(d, H, Ar-H), 7.09(d, H, Ar-H), 6.82 (d, H, Ar-H), 6.80(s, H, Ar-H), 3.85(s, 2H, -NH), 3.73(s, 3H, O-CH$_3$), 3.61(q, 4H, N-CH$_2$-), 1.23(s, 9H, N$^+$-CH$_3$), 1.19(t, 6H, -CH$_2$-CH$_3$); Anal. Calcd. for $C_{30}H_{34}Cl_2N_{10}OS$: C, 55.13%; H, 5.24%; N, 21.43%. Found: C,

55.06%; H, 5.44%; N, 21.82%.

D-4, the yield was 93.59%, FT-IR (v cm^{-1}): 3326, 2960, 2918, 2870, 2845, 2776, 1721, 1659, 1601, 1529, 1481, 1405, 1366, 1163, 1114, 967, 792; ^{1}H-NMR (DMSO-d_6, δ_H, ppm): 8.61(t, 2H, Ar-H), 8.56(t, H, Ar-H), 8.16(t, 2H, Ar-H), 7.67(d, H, Ar-H), 7.60(s, H, Ar-H), 7.12(d, H, Ar-H), 7.09(d, H, Ar-H), 6.78(d, H, Ar-H), 6.75(s, H, Ar-H), 4.82(m, 2H, NH-CH_2-), 4.22(t, 2H, -N^+-CH_2), 3.90(s, 2H, -NH), 3.86(s, 3H, O-CH_3), 3.56(q, 4H, N-CH_2-), 1.23(t, 6H, -CH_2-CH_3); Anal. Calcd. for $C_{28}H_{30}Cl_2N_{10}OS$: C, 53.76%; H, 4.83%; N, 22.39%. Found: C, 53.69%; H, 4.95%; N, 22.45%.

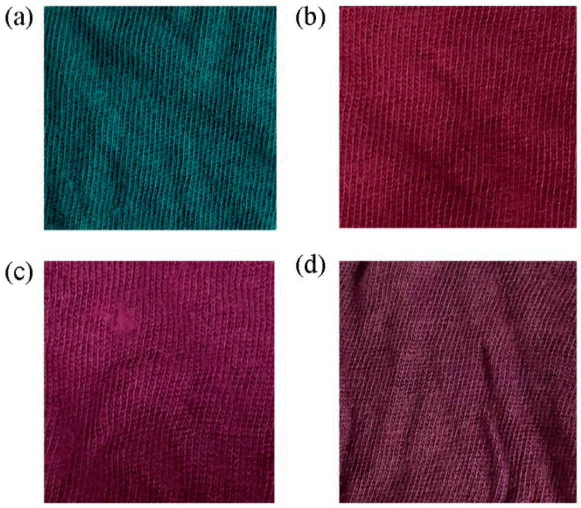

Fig.S2 Digital pictures for acrylic fabric (1%owf)
(a)D-1; (b)D-2; (c)D-3; (d)D-4

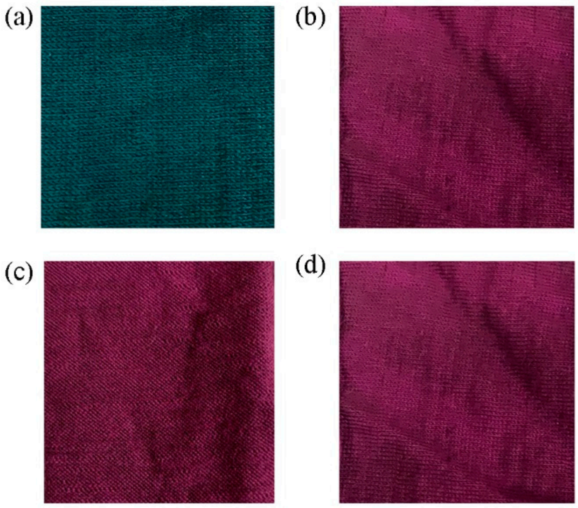

Fig.S3 Digital pictures for wool fabric (1%owf)
(a)D-1; (b)D-2; (c)D-3; (d)D-4

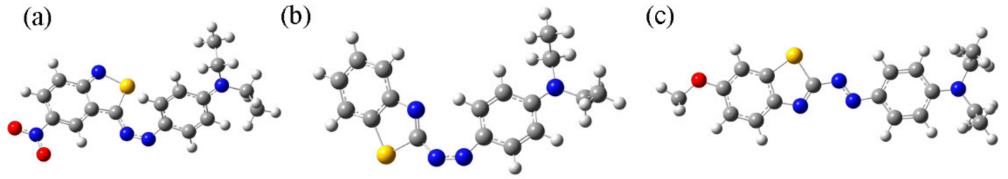

Fig.S4 Optimized structure for the chromophore in reactive dyes (ball & bond type)
